# miR-133a Regulates Adipocyte Browning In Vivo

**DOI:** 10.1371/journal.pgen.1003626

**Published:** 2013-07-11

**Authors:** Weiyi Liu, Pengpeng Bi, Tizhong Shan, Xin Yang, Hang Yin, Yong-Xu Wang, Ning Liu, Michael A. Rudnicki, Shihuan Kuang

**Affiliations:** 1 Department of Animal Sciences, Purdue University, West Lafayette, Indiana, United States of America; 2 Regenerative Medicine Program, Ottawa Hospital Research Institute, Ottawa, Ontario, Canada; 3 Program in Gene Function and Expression, University of Massachusetts Medical School, Worcester, Massachusetts, United States of America; 4 Department of Molecular Biology, University of Texas Southwestern Medical Center, Dallas, Texas, United States of America; 5 Purdue University Center for Cancer Research, Purdue University, West Lafayette, Indiana, United States of America; University of California San Francisco, United States of America

## Abstract

Prdm16 determines the bidirectional fate switch of skeletal muscle/brown adipose tissue (BAT) and regulates the thermogenic gene program of subcutaneous white adipose tissue (SAT) in mice. Here we show that miR-133a, a microRNA that is expressed in both BAT and SATs, directly targets the 3′ UTR of *Prdm16*. The expression of miR-133a dramatically decreases along the commitment and differentiation of brown preadipocytes, accompanied by the upregulation of Prdm16. Overexpression of miR-133a in BAT and SAT cells significantly inhibits, and conversely inhibition of miR-133a upregulates, *Prdm16* and brown adipogenesis. More importantly, double knockout of *miR-133a1* and *miR-133a2* in mice leads to elevations of the brown and thermogenic gene programs in SAT. Even 75% deletion of miR-133a (*a1^−/−^a2^+/−^*) genes results in browning of SAT, manifested by the appearance of numerous multilocular UCP1-expressing adipocytes within SAT. Additionally, compared to wildtype mice, *miR-133a1^−/−^a2^+/−^* mice exhibit increased insulin sensitivity and glucose tolerance, and activate the thermogenic gene program more robustly upon cold exposure. These results together elucidate a crucial role of miR-133a in the regulation of adipocyte browning in vivo.

## Author Summary

Global obesity and associated health issues have raised the significance of adipocyte biology. Adipose tissues are classified as brown and white adipose. White adipose tissues store lipids, leading to overweight, obesity, insulin resistance and Type2 diabetes. In contrast, brown adipose tissues use lipid storage to generate heat, increase insulin sensitivity, and are negatively correlated with the incidence of Type2 diabetes. Recent studies indicate that white adipose is plastic and contains an intermediate type of adaptive adipocytes (so-called beige/brite adipocytes) that have the energy-dissipating properties of brown adipocytes. Prdm16 is a key molecule that determines the development of both brown and beige adipocytes. Thus, Prdm16 represents a novel molecular switch that expands brown/beige adipocytes and increases energy expenditures. However, how Prdm16 is regulated has been unclear. Here we report that the microRNA miR-133a specifically targets Prdm16 at the posttranscriptional level. Inhibition or knockout of miR-133a significantly increases Prdm16 expression and the thermogenic gene program in white adipose tissues, resulting in dramatically enhanced insulin sensitivity in animals. Our results suggest that miR-133a represents a potential drug target against obesity and Type2 diabetes.

## Introduction

Adipose tissues are classified as brown (BAT) and white (WAT), and an intermediate category of “brite” or “beige” adipocytes exist within subcutaneous WAT [1], [2]. WAT are located in multiple subcutaneous or visceral locations of body, in the form of distinct fat depots, and contribute to overweight, obesity, insulin resistance and Type 2 diabetes. Brown adipocytes contain more mitochondria and express high levels of Ucp1, a mitochondria inner-membrane channel uncoupling ATP production with oxidative phosphorylation, therefore producing heat and dissipating chemical energy. Due to the global concern of obesity, browning of the white adipocytes, the induction of white adipocytes into beige adipocytes, has become a research focus. The signaling pathway that determines the developmental commitment and differentiation of brown and beige adipocytes is therefore crucial for understanding the process and importance of adipose browning.

Prdm16 is a critical regulator of brown adipocyte development and determines the thermogenic gene program in SAT. Downregulation of Prdm16 in brown adipocytes promotes their fate switch to myoblasts [3]. Conversely, ectopic overexpression of Prdm16 and its co-activator C/EBPβ in myoblasts or fibroblasts transdifferentiated them into brown adipocytes [3], [4]. Similarly, overexpression of Prdm16 in the stromal vascular fraction (SVF) cells of SAT led to the browning of white adipocytes [5]. Mechanistic studies have shown that Type 2 diabetic drug Rosiglitazone can stabilize Prdm16 protein, which activates PPARγ2 and initiates a brown adipocyte gene program that converts white adipocytes to beige adipocytes [6]. The signals that regulate Prdm16 transcription and post-transcriptional modification may offer new strategies for clinical applications and drug discoveries.

MicroRNAs are small non-coding RNAs that negatively regulate mRNA stability or protein translation through targeting the 3′untranslated regions (UTR) of mature mRNA. Previous studies have demonstrated that several myogenic microRNAs (i.e. miR-1, miR-206 and miR-133) are enriched in BAT in relative to WAT [7]. In addition, the cluster of miR-193b and miR-365, downstream signals of Prdm16, are required for brown adipocyte differentiation [8]. Moreover, miR-196a mediates the browning of white adipocytes through targeting Hoxc8, a repressor of brown adipogenic marker C/EBPβ [9]. These studies indicate that microRNAs play important roles in brown adipose development and the browning of white adipocytes.

In the present study, we examined the expression of over 30 microRNAs in the anterior subcutaneous WAT (asWAT) and inguinal WAT (ingWAT) that expressed relatively low and high levels of Prdm16, respectively. We identified several microRNAs whose expression is inversely correlated to Prdm16 expression. Based on this discovery, we conducted gain- and loss- of function studies to demonstrate that miR-133a regulates brown adipocyte biogenesis and browning of white adipocytes through the repression of Prdm16. Analysis of miR-133a knockout mice confirmed the in vivo function of this microRNA in regulating the adaptive plasticity of white adipocytes. We conclude that miR-133a plays a repressive role in adipocyte browning.

## Results

### miR-133a targets the 3′ UTR of Prdm16

**Figure 1:**
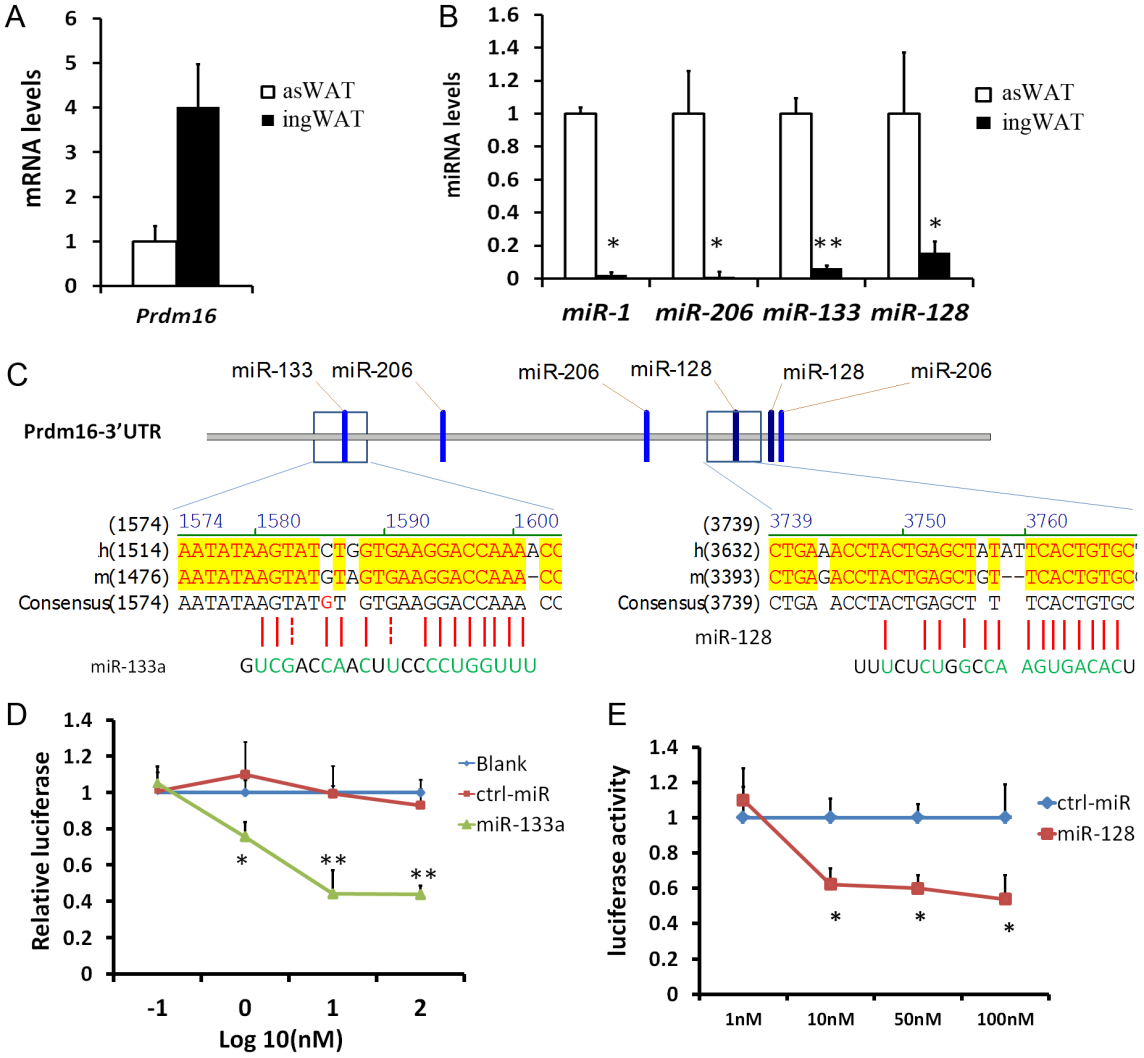
miR-133a and miR-128 target the 3′ UTR of Prdm16 in HEK293 cells. (A–B) qPCR analysis of miR-1, miR-206, miR-133a, miR-128 and Prdm16, for asWAT and ingWAT of wildtype mice. (C) Putative miRNA target sites within the 3 ′UTR of *Prdm16*. (D–E) Luciferase assay. Plasmids carrying luciferase gene linked to 3′ UTR of *Prdm16* were cotransfected to HEK293 cells, along with control miRNA, miR-133a (D) or miR-128 (E) at indicated doses. Luciferase activity was measure at 48h post-transfection and normalized. N = 4, *P<0.05, **P<0.01.

In the course of adipogenic marker screening among SAT depots, we found that *Prdm16* is expressed at much higher levels in the ingWAT compared to the asWAT (Fig. 1A). Interestingly, we identified four miRNAs (miR-1, miR-206, miR-133a and miR-128) that are expressed at significantly lower levels in the ingWAT compared to the asWAT (Fig. 1B, Fig. S1). The strong inverse correlation between the expression of Prdm16 and miRNAs implies that *Prdm16* may be regulated by these miRNAs. Within the 3′ UTR of *Prdm16*, there are putative target sites for miR-1, miR-206, miR-133a and miR-128 (Fig. 1C), raising the possibility that these miRNAs target *Prdm16* mRNA. Using classical luciferase assay in HEK293 cells, we found that miR-133a indeed repressed the luciferase activity by 20% at 1 nM and over 50% at 10–100 nM (Fig. 1D). Mutation of the miR-133a target sequence in the 3′ UTR of *Prdm16* totally abolished the repression of luciferase activity by miR-133a (data not shown). miR-128 also repressed the luciferase activity by ∼50% at 10–100 nM (Fig. 1E). Both miR-1 and miR-206 failed to repress the luciferase activity. These results suggest that miR-133a and miR-128 targets the 3′ UTR of *Prdm16*.

### Downregulation of miR-133a is accompanied by upregulation of *Prdm16* during brown adipocyte commitment and differentiation

**Figure 2:**
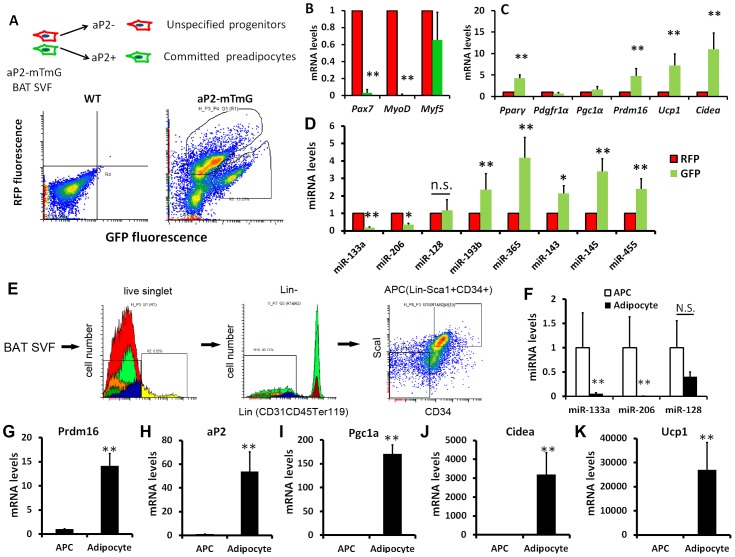
Downregulation of miR-133a with upregulation of *Prdm16* along brown adipocyte commitment and differentiation. (A) In aP2-Cre/mTmG mouse model, aP2 derived cells show green fluorescence (mG^+^) and non-aP2 derived cells show red fluorescence (mT^+^). The stromal-vascular fraction (SVF) of BAT was sorted based on fluorescence and the freshly sorted cells were used for qPCR analysis of (B) myogenic markers, (C) adipogenic markers and (D) adipogenic miRNAs. (E) Strategy for isolating adipose progenitor cells (APC) by FACS. (F) Relative expression of miR-113a, miR-206 and miR-128 in freshly sorted APC and mature adipocytes collected from the floating fraction of collagenase digested BAT. (G–K) Relative expression of BAT related genes in freshly sorted APC and mature adipocytes. N = 3–5, *P<0.05, **P<0.01.

Prdm16 is a transcriptional regulator that controls brown adipocyte fate determination [3]. As miR-133a targets the 3′ UTR of *Prdm16*, we sought to examine if miR-133a is involved in Prdm16-mediated brown adipocyte commitment and differentiation. To separate committed preadipocytes from more primitive progenitors in the SVF of BAT, we used the aP2-Cre/mTmG mouse model, in which aP2 lineage cells show green fluorescence (mG^+^) and non-aP2 derived cells exhibit red fluorescence (mT^+^). Previous studies reported that aP2 expression marks adipocyte progenitors but not bipotential stem cells [10], [11], [12]. The SVF cells of BAT was sorted based on mT and mG fluorescence and subjected to gene expression analyses (Fig. 2A).

Compared to the non-committed (mT^+^) cells, committed (mG^+^) preadipocytes completely lost the expression of *Pax7* and *MyoD*, two myogenic genes expressed by BAT, though the expression of dual BAT/muscle marker *Myf5* was the same in both populations (Fig. 2B). These data indicate that committed preadipocytes concomitantly downregulate the expression of myogenic genes. The adipogenic commitment of mG^+^ cells is further supported by the elevated expression of the adipogenic markers, including *Pparγ2*, *Prdm16*, *Ucp1* and *Cidea*, compared to the mT^+^ cells (Fig. 2C). Notably, qPCR results demonstrate that miR-133a is decreased by 80%, but miR-128 is not significantly altered, in the mG^+^ cells (Fig. 2D), suggesting that miR-133a is more likely to target Prdm16 in vivo. By contrast, the expression of miR-193b and miR-365, previously shown to be direct downstream targets of Prdm16 and required for BAT differentiation [8], were increased in mG^+^ compared to mT^+^ cells (Fig. 2D). The mG^+^ cells also expressed higher levels of miR-143, miR-145 and miR-455 (Fig. 2D), known as adipogenic miRNAs [7]. We further compared the relative expression of miRNAs and BAT related genes in adipose progenitor cells (APC, Fig. 2E) and mature adipocytes collected from the floating fractions of enzymatically digested BAT. Consistently, miR-133a but not miR-128 was significantly downregulated in the differentiated mature adipocytes compared to APC (Fig. 2F). By striking contrast, *Prdm16* and other adipogenic markers including *aP2*, *Pgc1α*, *Cidea* and *Ucp1* were all dramatically upregulated in mature adipocytes (Fig. 2G–K). Together, these data indicate that miR-133a downregulation along the commitment and differentiation of brown adipocytes might play a role in Prdm16 upregulation during brown adipogenesis.

### miR-133a inhibits brown adipocyte biogenesis in BAT and SAT

**Figure 3:**
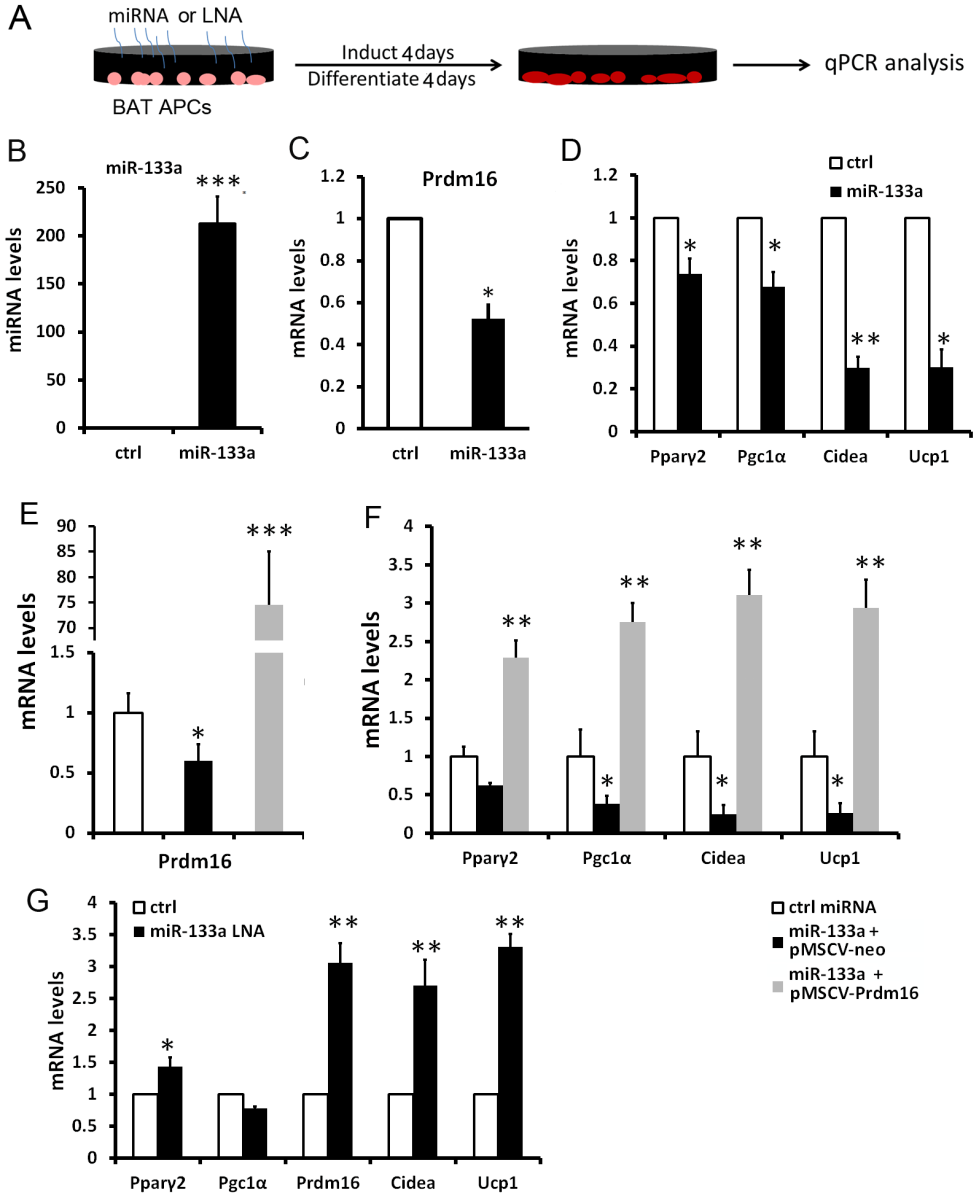
miR-133a inhibits brown adipocyte biogenesis of BAT progenitors. (A) Strategies for electroporation of miRNAs or LNAs to cultured BAT APCs, followed by induction and differentiation for 4 days. (B–D) qPCR analysis of miR-133a and the brown markers in the control and miR-133 overexpressed BAT adipocytes. (E–F) qPCR analyses of Prdm16 and BAT specific genes in adipocytes overexpressing control miRNA, miR-133a with control retrovirus and miR-133a with Prdm16-overexpressing retrovirus. (G) qPCR analysis of BAT marker gene expression after miR-133a was inhibited by LNAs. N = 3, *P<0.05, **P<0.01, ***P<0.001.

To examine if miR-133a directly regulates Prdm16 and plays a role in BAT adipogenesis, we overexpressed miR-133a in cultured BAT APCs (Fig. 3A). Electroporation-mediated gene transfer resulted in 213-fold increase in the expression of miR-133a (Fig. 3B). As a consequence, *Prdm16* mRNA was downregulated by 48% (Fig. 3C), and the BAT markers *Ucp1* and *Cidea* were downregulated by ∼70% (Fig. 3D). Other adipogenic genes *Pparγ2* and *Pgc1α* were moderately decreased, by 25%∼30% (Fig. 3D). Importantly, the effects of miR-133a overexpression were totally reversed by concomitant overexpression of *Prdm16*, and even led to ∼3 fold increase (overshoot) in adipogenic marker expression (Fig. 3D–E). This complete reversal and overshoot can be explained as overexpression of the miR-133a insensitive *Prdm16* cDNA (lacking 3′ UTR) overrides the repression of miR-133a on endogenous *Prdm16*. Conversely, we used antisense oligonucleotide LNAs to specifically inhibit miR-133a in BAT APCs. Downregulation of miR-133a led to 40% upregulation of *Pparγ2 and* ∼3-fold increases of *Prdm16*, *Ucp1*, and *Cidea* (Fig. 3G). Our data suggest that BAT adipogenesis is inhibited by overexpression, and promoted by inhibition, of miR-133a.

Similarly, we overexpressed miR-133a in SAT preadipocytes (Fig. S2A). A 95-fold overexpression of miR-133a led to 38% downregulation of *Prdm16* (Fig. S2B), accompanied by 50% downregulation of *Pparγ2* and ∼70% downregulation of *Ucp1*, *Cidea* and *Lhx8* (Fig. S2C). Our results together suggest that miR-133a represses BAT adipogenesis and WAT browning through targeting *Prdm16*.

### Genetic ablation of *miR-133a* upregulates the thermogenic gene program in SAT

**Figure 4:**
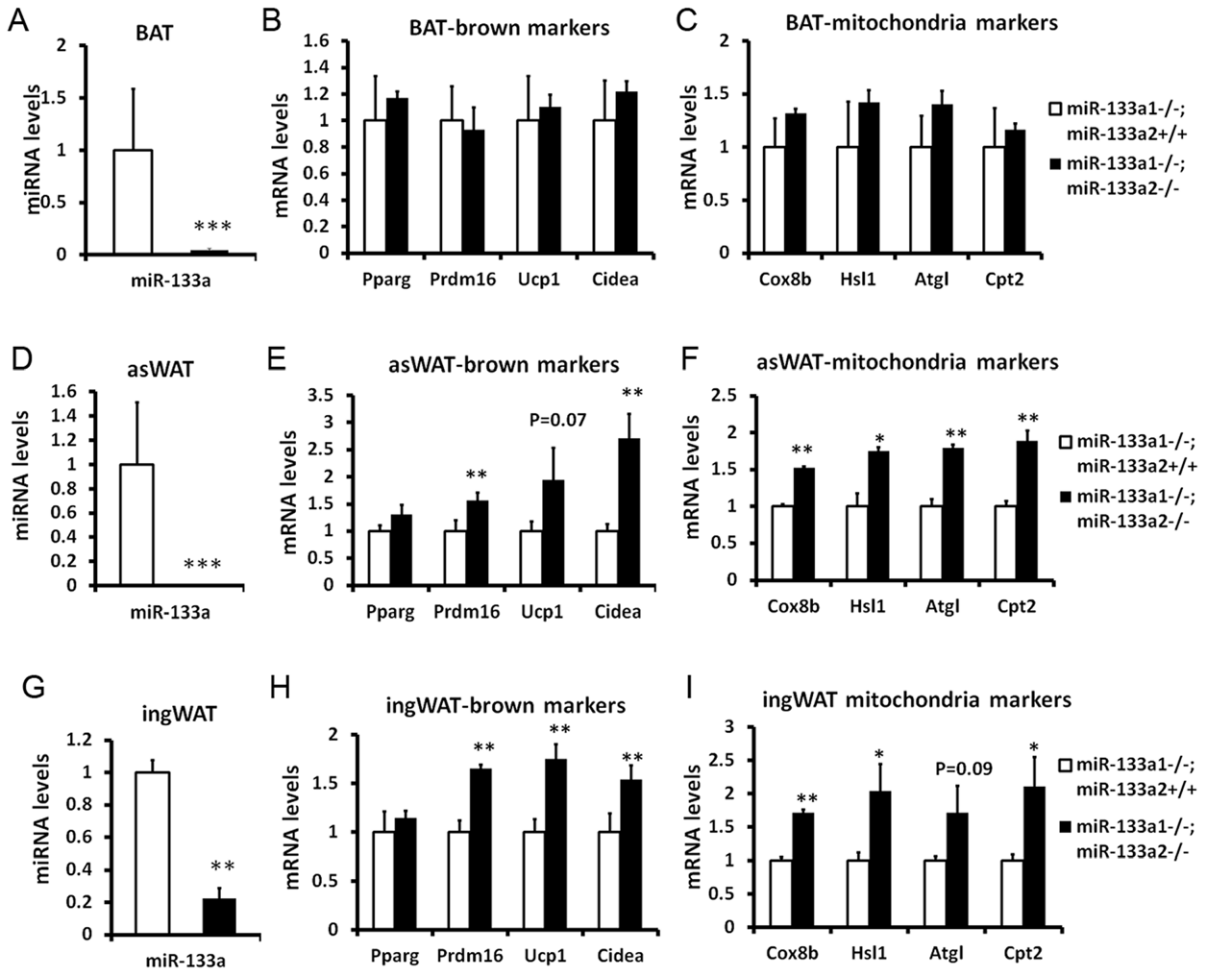
Genetic ablation of *miR-133a* promotes the browning and thermogenic gene program in SAT but not BAT. miR-133a has two alleles, miR-133a1 and miR-133a2. The miR-133a-dKO mice (miR-133a1^−/−^; miR-133a2^−/−^) were obtained by intercrossing mice with the genotype of miR-133a1^−/−^; miR-133a2^+/−^. The mice (miR-133a1^−/−^; miR-133a2^−/−^) were used as a control for the qPCR analysis of BAT, asWAT and ingWAT. (A) miR-133a levels in BAT, (B) brown marker expression in BAT, (C) thermogenic gene program in BAT. (D) miR-133a levels in asWAT, (E) brown marker expression in asWAT, (F) thermogenic gene program in asWAT. (G) miR-133a levels in ingWAT, (H) brown marker expression in ingWAT, (I) thermogenic gene program in ingWAT. The brown markers include *Prdm16*, *Ucp1* and *Cidea*; the thermogenic genes include *Cox8b*, *Hsl*, *Atgl* and *Cpt2*. N = 3, *P<0.05, **P<0.01.

miR-133a has two alleles, *miR-133a1* and *miR-133a2*, which have identical sequences and are located in different chromosomes. To investigate the function of miR-133a in BAT and SAT, we examined miR-133a double knockout mice (dKO, *miR-133a1^−/−^a2^−/−^*), generated by intercrossing mice with the genotype of *miR-133a1^−/−^a2^+/−^*. Previous study indicates that compared to wildtype mice, knockout of either *miR-133a1* or *miR-133a2* led to a 40%–50% downregulation of miR-133a in skeletal and cardiac muscles [13], [14]. We examined the expression of various BAT and mitochondria markers in the dKO mice using *miR-133a1^−/−^a2^+/+^* littermates as the control. As expected, miR-133a levels were reduced by 80%–98% in BAT, asWAT and ingWAT in the dKO compared to the controls (Fig. 4A, 4D, 4G).

Surprisingly, neither BAT markers (*Prdm16*, *Ucp1* and *Cidea*) nor mitochondria and lipolysis markers (*Cox8a*, *Hsl*, *Atgl* and *Cpt2*) were significantly affected in BAT tissue of miR-133a dKO mice (Fig. 4B–C). In striking contrast, the dKO mice had ∼1.5–2 fold elevated expression of the brown adipose markers including *Prdm16*, *Ucp1* and *Cidea*, and the mitochondria/lipolysis markers including *Cox8b*, *Hsl*, *Atgl* and *Cpt2*, both in asWAT (Fig. 4E and 4F) and ingWAT (Fig. 4H and 4I). As SAT is responsible for cold- and hormone-induced browning, these data suggest that miR-133a represses browning of white adipocytes in vivo under physiological conditions.

### Reduced level of miR-133a leads to browning of WAT and improves body insulin sensitivity in vivo

Due to high perinatal lethality (76%) and cardiac myopathy-related postnatal sudden death of the few surviving miR-133a dKO mice [13], [14], we used in the subsequent studies *miR-133a1^−/−^a2^+/−^* mice that had three out of the four miR-133a alleles knocked out but had normal cardiac and skeletal muscles. We used age, gender and genetic background matched WT mice (*miR-133a1^+/+^a2^+/+^*) as control. We reasoned that if we can detect phenotype in mice with 75% reduction of miR-133a, then there should be even more robust effects if miR-133a is completely knocked out.

**Figure 5:**
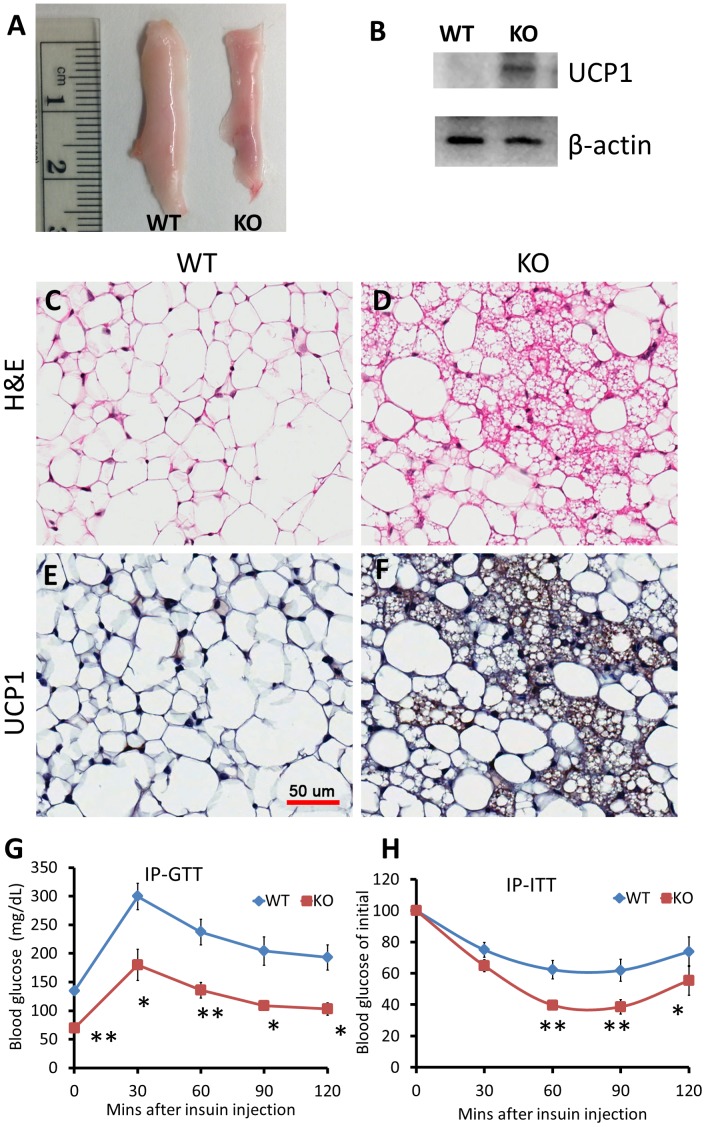
Knockdown of miR-133a leads to browning of WAT and improves body insulin sensitivity in vivo. Wildtype (WT; miR-133a1^+/+^a2^+/+^) and miR-133a knockdown (KO; *miR-133a1^−/−^a2^+/−^*) mice were used. (A) Representative image of inguinal WAT isolated from WT and KO mice. (B) Western blot image showing relative expression of UCP1 and β-actin in WT and KO ingWAT. (C–D) H&E staining of WT (C) and KO (D) ingWAT. (E–F) immunohistostaining of UCP1 of WT (E) and KO (F) ingWAT. UCP1 signal is in brown and cell membrane is counter stained in blue. Scale bar = 50 µm. (G) Blood glucose levels during IP-GTT test (n = 4 pairs of mice). (H) Blood glucose levels after IP insulin injection, normalized to initial glucose measurement as 100% (n = 6 pairs of mice). *P<0.05, **P<0.01.

To examine if the observed upregulation of brown adipose and mitochondrial specific genes in SAT of miR-133a mutants are associated with browning of white adipose, we conducted histological analysis. The ingWAT of *miR-133a1^−/−^a2^+/−^* mice appeared to be browner than that of wildtype mice (Fig. 5A). Western blots confirm that UCP1 protein is indeed upregulated in the ingWAT of *miR-133a1^−/−^a2^+/−^* mice compared to the wildtype mice (Fig. 5B). H&E staining reveals the appearance of numerous multilocular brown adipocyte-like cells in the ingWAT of *miR-133a1^−/−^a2^+/−^* mice, but not wildtype mice (Fig. 5C–D). Immunohistochemical staining with brown adipocyte specific UCP1 antibody indicates that these multilocular brown adipocytes are UCP1^+^ (brown signal), and the UCP1 immunoreactivity is much more abundant in the ingWAT of *miR-133a1^−/−^a2^+/−^* mice compared to the wildtype mice (Fig. 5E–F).

To directly test how miR-133a affects insulin sensitivity and glucose metabolism, we conducted glucose tolerance test (GTT) and insulin tolerance test (ITT). Strikingly, GTT indicates that the miR-133a mutants had ∼50% lower overnight fasting glucose levels than WT mice (Fig. 5G). The mutants also had much improved glucose tolerance at all the time points examined (Fig. 5G). Similar results were observed by ITT. Upon I.P. administration of insulin (0.75 U/Kg BW), blood sugar dropped much more rapidly and remained lower during 2 h examination period in the *miR-133a1^−/−^a2^+/−^* compared to the wildtype mice (Fig. 5H). These results indicate that reduced miR-133a level is associated with improved insulin sensitivity and glucose disposal in vivo.

### Reduced level of miR-133a promotes the activity of cold-inducible thermogenesis gene program in vivo

**Figure 6:**
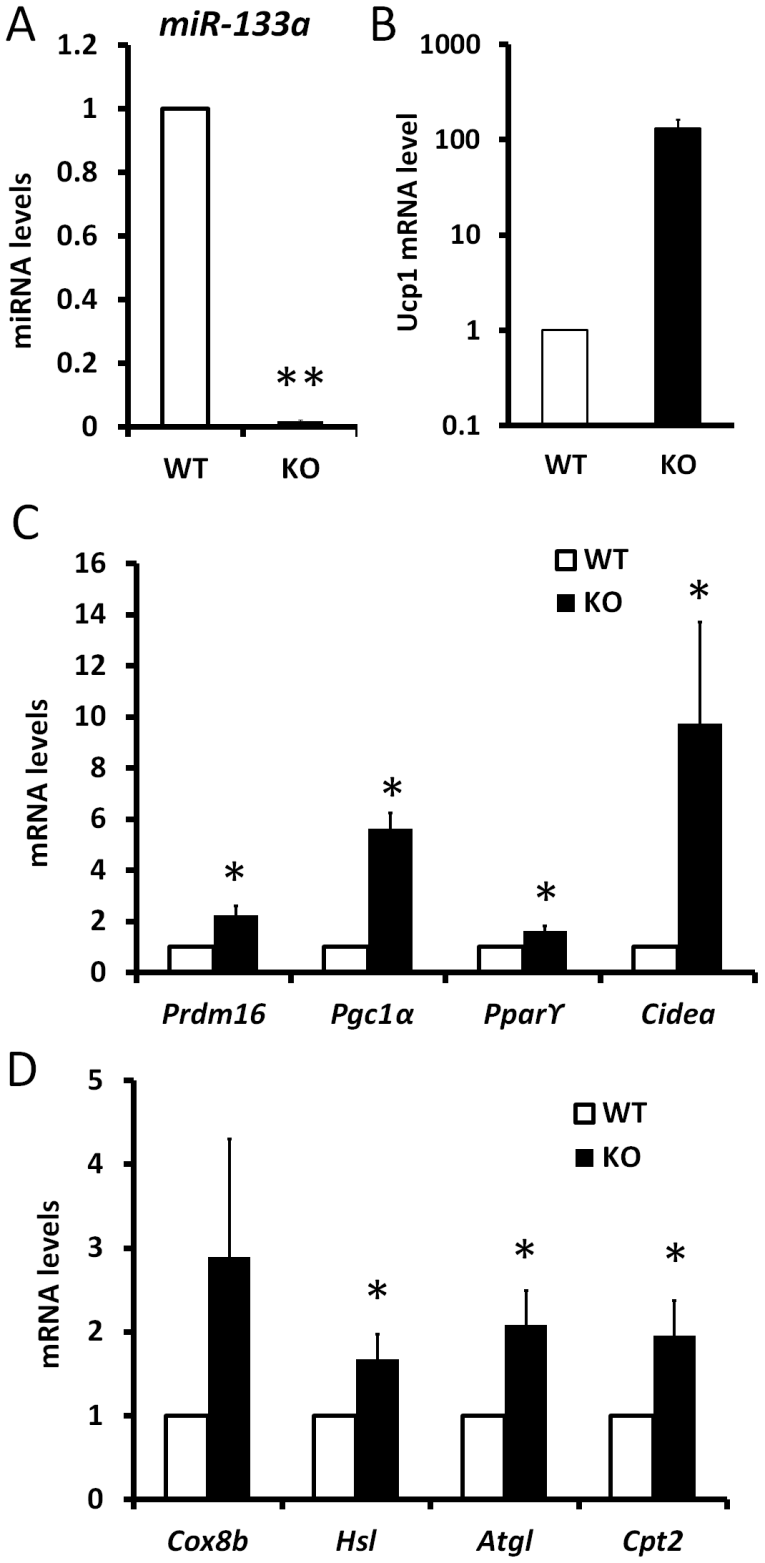
Knockdown of miR-133a promotes the activity of cold-inducible thermogenesis gene program in vivo. qPCR analysis of relative expression of genes in inguinal WAT tissue after 5 day exposure of wildtype (WT) and miR-133a knockdown (KO; *miR-133a1^−/−^a2^+/−^*) mice at 4°C. (A) miR-133a, (B) *Ucp1*, (C) other brown adipocyte markers, *Prdm16, Pgc1α, Pparγ2, Cidea*, (D) mitochondria genes *Cox8b* and *Cpt2*, and lipolysis genes *Hsl* and *Atgl*. N = 4–6, *P<0.05, **P<0.01.

Subcutaneous white adipose is capable of thermogenesis under cold exposure. The adaptive thermogenesis capacity is correlated to the level of Prdm16 expression. To investigate if inhibition of miR-133a promotes the adaptive thermogenesis of white adipose, we exposed *miR-133a1^−/−^a2^+/−^* and wildtype mice to cold environment. After 5 d exposure at 4°C, the level of miR-133a in *miR-133a1^−/−^a2^+/−^* ingWAT is about 2% of that in wildtype ingWAT (Fig. 6A), but the level of Ucp1 is about 130 times higher in *miR-133a1^−/−^a2^+/−^* ingWAT (Fig. 6B). The expression levels of *Pparγ2*, *Prdm16*, *Pgc1a*, and *Cidea* genes were 1.6-, 2.5-, 5-, and 10-fold higher in the ingWAT of *miR-133a1^−/−^a2^+/−^* mice compared to wildtype mice (Fig. 6C). Accordingly, genes related to mitochondrial function (*Cox8b* and *Cpt2*) and lipolysis (*Hsl* and *Atgl*) were also upregulated in the *miR-133a1^−/−^a2^+/−^* ingWAT (Fig. 6D). Consistent with the relative mRNA levels, UCP1 protein levels in asWAT and ingWAT of the *miR-133a1^−/−^a2^+/−^* mice are obviously higher than those of the wildtype mice at room temperature and after cold exposure (Fig. S3). Therefore, reduced level of miR-133a promotes the activity of cold-inducible thermogenesis gene program in vivo.

### Reduced level of miR-133a predispose white preadipocytes to become adaptive beige adipocytes upon differentiation

**Figure 7:**
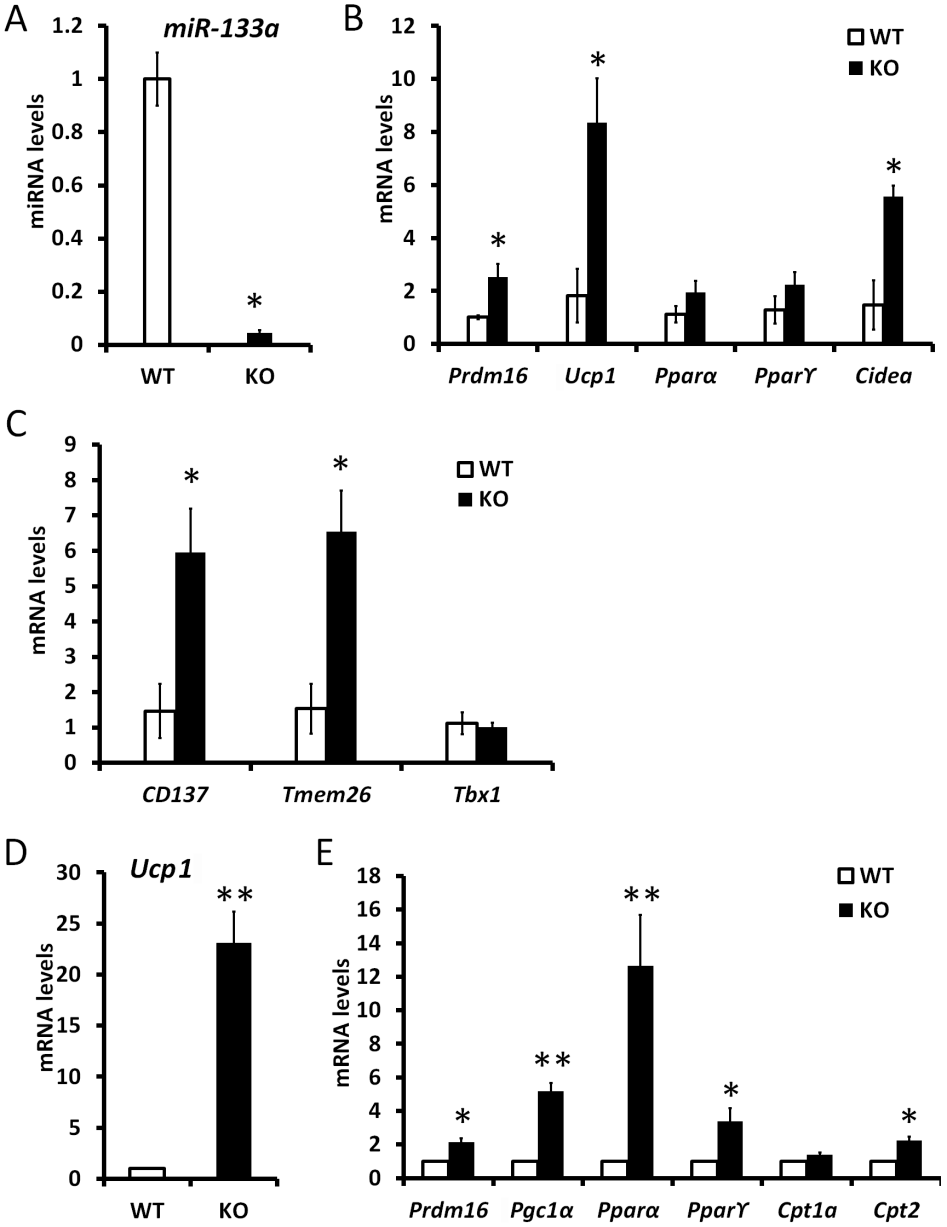
Mutation of miR-133a predispose white preadipocytes to become adaptive beige adipocytes upon differentiation. qPCR analysis of relative gene expression in inguinal WAT tissue (n = 3 pairs) and cultured WAT adipocytes from SVF (n = 4 pairs) of wildtype (WT) and miR-133a knockdown (KO; *miR-133a1^−/−^a2^+/−^*) mice. (A) miR-133a in ingWAT, (B) BAT marker genes in ingWAT, (C) beige adipocyte marker genes, *CD137*, *Tmem26* and *Tbx1*, in ingWAT. (D) *Ucp1* and (E) other BAT marker gene expression in adipocytes differentiated from stromal vascular cells of ingWAT. *P<0.05, **P<0.01.

The adaptive thermogenesis of subcutaneous WAT has been shown to be mediated by a population of beige adipocytes [15]. We examined the ingWAT of *miR-133a1^−/−^a2^+/−^* and wildtype mice to address if reduction of miR-133a predisposes white preadipocytes to become beige cells that express unique beige markers and common BAT markers [15]. The expression level of miR-133a is reduced by 95% in the ingWAT of *miR-133a1^−/−^a2^+/−^* mice compared to the wildtype mice (Fig. 7A). Accordingly, the BAT-specific genes *Prdm16*, *Cidea* and *Ucp1* were expressed at 2.5-, 5.5- and 8.4-fold in the ingWAT of *miR-133a1^−/−^a2^+/−^* mice compared to the wildtype mice (Fig. 7B). Importantly, beige adipocyte specific *CD137* and *Tmem26* genes were also expressed at higher levels (∼6 times) in the *miR-133a1^−/−^a2^+/−^* ingWAT compared to the wildtype (Fig. 7C). We further isolated and differentiated stromal vascular preadipocytes from subcutaneous WAT of *miR-133a1^−/−^a2^+/−^* and wildtype mice. Adipocytes differentiated from the *miR-133a1^−/−^a2^+/−^* preadipocytes expressed 23 times more *Ucp1* than wildtype adipocytes (Fig. 7D). Accordingly, expression of other BAT-specific genes *Prdm16*, *Pgc1α*, *Pparα*, *Pparγ2*, and *Cpt2* were also upregulated in the in vitro differentiated *miR-133a1^−/−^a2^+/−^* adipocytes (Fig. 7E). Together, the in vivo and in vitro gene expression analysis demonstrate that inhibition of *miR-133a* predispose white preadipocytes to become adaptive beige adipocytes upon differentiation.

## Discussion

We identified miR-133a as a regulator of Prdm16 in vivo. Based on the mutual exclusion model of miRNA-mRNA interactions, the cells that express high levels of miRNAs should have less expression of their targets, and vice versa [16]. However, we found both miR-133a and Prdm16 are expressed at very high levels in BAT compared to WAT. This paradox led us to hypothesize that within the BAT, there are different populations of cells that express high levels of miR-133a or Prdm16, respectively, with the notion that miR-133a is highly expressed in cells expressing low levels of Prdm16, and vice versa. In the course of brown adipocyte commitment and differentiation, aP2 expression marks more committed progenitors and preadipocytes, whereas aP2^−^ cells contain more primitive adipocyte progenitors, mesenchymal stem cells and other cell types [12]. Compared to the more primitive cells (aP2^−^), aP2^+^ cells express increased Prdm16 and decreased miR-133a. Orchestrated with this notion is the observation that compared to APCs, differentiated brown adipocytes nearly lost the expression of miR-133a. These data imply that miR-133a-mediated Prdm16 repression occurs mainly in uncommitted stem cells to restrict their differentiation towards brown fat, and maintain their multipotency. The luciferase reporter assay, gain- and loss-of-function studies provide direct evidence that miR-133a target Prdm16. In consistency with our study, two recent studies demonstrated that miR-133 can target Prdm16 in both satellite cells and brown adipose cell lines [17], [18].

Interestingly, miR-133a dKO mouse has adipocyte browning in SAT but has no overt phenotype in BAT. Several possibilities might have led to this observation. First, miR-133b, another miR-133 family member, is also highly expressed in BAT (than in WAT) and maintains its expression in the miR-133a dKO BAT. Notably, miR-133b is dramatically downregulated in the asWAT and ingWAT of miR-133a dKO mice for unknown reasons. The loss of both miR-133a and miR-133b in SAT might have led to the upregulation of Prdm16 and activation of the BAT and thermogenic gene program. Second, the loss of miR-133a may be insufficient to further upregulate Prdm16, which is already highly expressed in the BAT. By contrast, Prdm16 is expressed at levels several fold lower in the SAT and the loss of miR-133a can therefore lead to an upregulation of Prdm16.

It has been reported that 76% of *miR-133a1^−/−^a2^−/−^* dKO mice die prior to P10 and the few surviving mice are subjected to sudden death due to cardiomyopathy [13]. Due to the extremely low survival rate of the *miR-133a1^−/−^a2^−/−^* dKO mice [13], [14], we used *miR-133a1^−/−^a2^+/−^* that had three out of the four miR-133a alleles knocked for most in vivo studies. We found that the ingWAT of *miR-133a1^−/−^a2^+/−^* mice contains numerous multilocular adipocytes that express UCP1. Our observation that beige adipocyte specific *CD137* and *Tmem26* genes are highly upregulated in the *miR-133a1^−/−^a2^+/−^* compared to the wildtype ingWAT suggests that the multilocular adipocytes in the *miR-133a1^−/−^a2^+/−^* WAT are probably the cold-inducible adaptive beige adipocytes. Consistent with this notion, we found that *miR-133a1^−/−^a2^+/−^* mice activate the thermogenic gene program much more robustly than the wildtype mice upon cold exposure. More importantly, ITT and GTT demonstrate that the *miR-133a1^−/−^a2^+/^* ^−^ mice exhibit increased sensitivity to insulin and glucose tolerance compared to WT controls. These results together provide strong in vivo evidence that miR-133a regulates the normal physiological function of adipose tissues.

Upon catecholamine hormone stimulations, WAT depots undergo lipid mobilization, a lipolysis process that hydrolyzes triglycerides of white adipocytes [19]. Free fatty acids released from white adipocyte lipolysis undergo beta-oxidation in the mitochondria of brown adipose tissue and provide energy. The Fatty acids also activate special Ppar*γ2* complex which directly activates Ucp1 expression and dissipates chemical energy [20]. Prdm16 is a Ppar*γ2* coactivator that drives the brown adipocyte gene program [3]. Here we showed that miR-133a inhibits white adipocyte browning, it would be interesting to study if miR-133a is involved in the repression of hormone stimulated adipocyte browning process.

Obesity has disrupted catecholamine signals, leading to excess fat accumulation and multiple metabolic diseases [19]. Prdm16 drives expression of the browning and thermogenic gene program [2], [5]. Overexpression of Prdm16 in adipose lineage resulted in large number of beige cell formation in SAT and more energy expenditure, which improved glucose metabolism and enhanced insulin sensitivity [2]. However, there is no report to show the anti-obese and anti-diabetic role of Prdm16, or adipocyte browning, in obese and diabetic background. It would be interesting to examine if overexpression of Prdm16 in the ob/ob mice or the db/db mice can ameliorate excessive fat accumulation and improve system insulin sensitivity. In this study we showed that miR-133a negatively regulates Prdm16 and miR-133a KO mice have dramatic phenotype including adipocyte browning, improved glucose metabolism and insulin sensitivity. Consistent with our observation, blockage of endogenous miR133 by antisense nucleotides in mice can greatly lower blood glucose levels [18]. It remains to be investigated if inhibition of miR-133a can increase system energy expenditure in the ob/ob and db/db background.

Interscapular brown adipose is detectable in the newborn humans to maintain body temperature but its mass gradually decreases in the postnatal life [21]. Recent studies have demonstrated that adult humans develop active brown adipocytes in response to cold exposure and the amount of BAT is inversely correlated with body weight [22], [23], [24]. Detailed molecular signature analysis suggested that the adult human brown adipocytes are more similar to murine beige cells, but not the classical interscapular BAT cells [15], [25]. Our study revealed that miR-133a represses white adipocyte browning and beige adipocyte formation in the mouse model. It remains to be investigated if miR-133a also plays a crucial role in the naturally occurred adipocyte browning in humans. As the increased beige adipocytes absorb more glucose and increase insulin sensitivity, it will be interesting to investigate if miR-133a could be a potent drug target for clinical purposes.

## Materials and Methods

### Animals

All procedures involving the use of animals were performed in accordance with the guidelines presented by Purdue University's Animal Care and Use Committee. *mTmG* and aP2-Cre mice were from Jackson Lab under stock# 007576 and 005069, respectively. miR-133a knockout mice were previously described [13]. For glucose tolerance test (GTT), 7-week-old mice were fasted overnight and injected with 1.5 mg/g glucose/body weight. For insulin tolerance test (ITT), 2–3 month old mice were fasted for 4 hours and injected with 0.75 U/Kg insulin/body weight. The blood glucose levels were monitored at 30 min intervals for 2 h with an ACCU-CHEK Active Blood Glucose System (Roche) using tail tip blood samples. For cold exposure, mice in their regular filter-top cages with double bedding and nesting materials were placed in ventilated plastic bins and housed in a 4°C room for 5 days.

### Primary adipocyte cultures

Primary adipocyte cultures were performed as previously reported [26]. Interscapular BAT and various WAT depots were collected, minced and digested with isolation buffer for proper time at 37°C on a shaker. The isolation buffer contains 123 mM NaCl, 5 mM KCl, 1.3 mM CaCl_2_, 5 mM Glucose, 100 mM HEPES, 4% BSA, 1%P/S and 1.5 mg/ml Collagenase I. The digestion was stopped with DMEM containing 2%FBS and 1% HEPES, filtered through 100 µm filters, and cells were pelleted at 450× *g* for 5 min. The cells were cultured in growth medium containing DMEM, 20% FBS, 2% HEPES and 1% P/S at 37°C with 5% CO2 for 3 days, and then fresh media was changed every 2 days. Upon confluence, cells were exposed to induction medium for 4 days and then differentiation medium for several days until adipocytes mature. The induction medium contains DMEM, 10% FBS, 2.85 µM insulin, 0.3 µM dexamethasone (DEXA) and 0.63 mM 3-isobutyl-1-methylxanthine (IBMX) (Sigma), and the differentiation medium contains DMEM, 10% FBS, 200 nM insulin and 10 nM T3.

### Luciferase assay

Plasmids carrying *Renila* luciferase gene linked to a fragment of Prdm16-3′UTR harboring miR-133a putative binding sites were cotransfected to HEK293 cells, along with control miRNA or miR-133a mimic (Invitrogen). The mutant 3′ UTR of Prdm16 was performed by mutagenesis of the miR-133a recognized sequences from GGACCAA into TTGGTCC. Samples were collected at 48 h post-transfection. Luciferase activity was measured with the use of the Dual Luciferase Assay System (Promega), and the relative luciferase activities were normalized to *firefly* luciferase. Plasmids carrying *firefly* luciferase gene linked to fragments of Prdm16-3′UTRs harboring the putative target sites of miR-206, miR-1, or miR-128 were co-transfected to HEK293 cells along with control miRNA, miR-206 mimic, miR-1 mimic, or miR-128 mimic (Invitrogen). The relative luciferase activities were normalized to *Renila* luciferase.

### Fluorescent activated cell sorting (FACS)

The stromal vascular fraction of adipose tissues was isolated as described above and cells were filtered through 30 µm sterile nylon mesh. The BAT SVF cells from aP2-mTmG are selected on the basis of fluorescence characteristics. mTmG adipocytes were used as a positive control for gating RFP+ cells. Cell debris and dead cells were removed by staining of dead cell dye. The adipose progenitor cells were sorted out from SVF cells of wildtype mice by antibodies against CD31-PE-Cy7, CD45-PE-Cy7, Ter119-PE-Cy7, CD34-FITC, and Sca1-Pacific blue (eBioscience). After sorting, cells were collected for RNA extraction right away or cultured in CO2 incubator at 37°C for differentiation or transfection experiments.

### microRNA transfection of BAT APCs and SAT SVF cells

The transfection was performed by Neon electroporation system (Invitrogen). Final concentration of 500 nM miRNA mimics or miRNA LNAs were incubated with the indicated cells (50,000–100,000 cells) on ice for 5 min and the electroporation is performed under 1150 voltage, 20 msec intervals and 2 pulses. The cells were then seed on 12-well plates. After 12 hours the transfection complex was replaced with fresh adipogenic induction medium. After 4 days of induction, the medium was replaced with adipogenic differentiation medium and the cells were collected for RNA analysis after an additional 4 day differentiation.

### Retrovirus production and infection

The plasmids pMSCV-Prdm16 or the empty vector was transfected by lipofactamine 2000, along with the packing vector pEco to 10-cm Hek293 cells. Freshly isolated 48 h supernatants containing retrovirus particles were filtered and mixed with 4 ug/ml Polybrene. The mixtures (1 ml) were added to each well of BAT APCs, which have been recovered for 8–12 hours after the electroporation of miR-133a. Fresh culture medium was added 12 hours later and was replaced by BAT induction medium after additional 12 hours. Induction and differentiation were same as described above.

### Quantitative realtime PCR (qPCR)

RNA was extracted and purified from mature adipocytes of adipose tissues or cell cultures with Trizol and contaminating DNA was removed with DNase I. Random hexamer primers were used to convert RNA into cDNA. For microRNA qPCR, multiple adenosine nucleotides were added to 3′ end of RNAs by E. coli DNA polymerase and cDNAs were synthesized with a specific RT primer [27]. QPCR was performed by using a light cycler 480 (Roche) machine for 40 cycles and the fold change for all the samples was calculated by 2^−ΔΔct^ methods. *18s* was used as housekeeping gene for mRNA expression analysis. 18s and U6 mRNA was used as housekeeping gene for microRNA expression analysis.

### Histology and immunohistochemistry

Serial sections of white fat were cut at 4 µm thick, de-paraffinized, and rehydrated through xylene, ethanol, and water by standard methods. For antigen retrieval, slides were submerged in 0.01 mol/L sodium citrate (pH 6.0) and heated to 96°C for 20 minutes in a laboratory microwave (PELCO). Immunohistochemistry was performed on a Dako Autostainer (Dako, Carpinteria, CA). Slides were incubated with 3% hydrogen peroxide and 2.5% normal horse serum (S-2012, Vector), followed by incubation with rabbit polyclonal anti-UCP-1 primary antibody (ab23841, Abcam) diluted 1∶200 in 2.5% normal horse serum (Vector, S-2012) for 60 minutes. Primary antibody binding was detected with an anti-rabbit horseradish peroxidase (HRP)–ImmPRESS Anti-Rabbit Ig (peroxidase) Polymer Detection Kit (MP-7401, Vector). Labeling was visualized with 3, 3′-diaminobenzidine (DAB) as the chromogen (SK-4105, Vector). Slides were counterstained with Harris hematoxylin (EK Industries, Joliet, IL) and whole slide digital images were collected at 20× magnification with an Aperio ScanScope slide scanner (Aperio, Vista, CA).

### Statistical analysis

The data are presented with mean ± standard error of the mean (SEM). P-values were calculated using two-tailed student's t-test. The ones with P-value less than 0.05 were considered as statistic significant.

## Supporting Information

Figure S1 Relative expression of miroRNAs in various subcutaneous WAT depots. asWAT, anterior subcutaneous WAT; bsWAT, back subcutaneous WAT; ingWAT, inguinal WAT. The expression of bsWAT is normalized to 1. N = 3. *P<0.05, **P<0.01.
(TIF)

Figure S2 miR-133a inhibits adipocyte browning in SAT. SAT SVFs were transfected with synthetic miRNA133a by electroporation and cultured to confluence, followed by adipogenic induction and differentiation for 4 days each. (A–C) qPCR analysis of miR-133a and the brown markers after cells were differentiated. N = 3, *P<0.05, **P<0.01.
(TIF)

Figure S3 Knockdown of miR-133a upregulates UCP1 expression. Depots of asWAT and ingWAT were harvested from widltype (WT) and miR-133a knockdown (KO; *miR-133a1^−/−^a2^+/−^*) mice that were housed at room temperature or at 4°C for 5 days. Pictured are representative Western Blot images showing the relative expression levels of UCP1. Beta-Actin is used as internal control for protein input.
(TIF)
