# Supplementary figures and images for: miR-133a Regulates Adipocyte Browning In Vivo
Source: PLoS Genet. 2013 Jul 11;9(7):e1003626. doi: 10.1371/journal.pgen.1003626 (PMC3708806; doi:10.1371/journal.pgen.1003626)

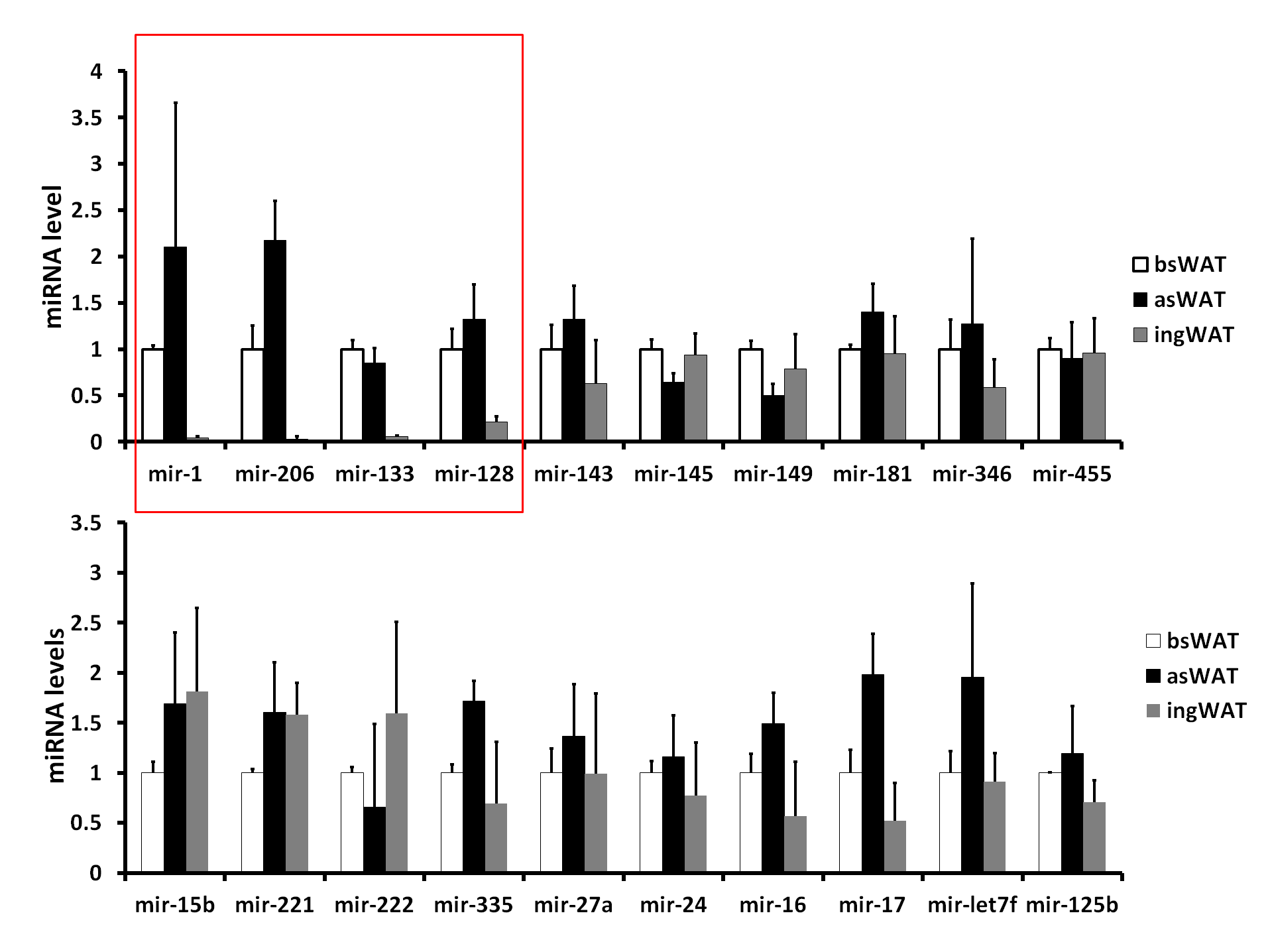

Supplement: Figure S1 — Relative expression of miroRNAs in various subcutaneous WAT depots. asWAT, anterior subcutaneous WAT; bsWAT, back subcutaneous WAT; ingWAT, inguinal WAT. The expression of bsWAT is normalized to 1. N = 3. *P<0.05, **P<0.01. (TIF) [file pgen.1003626.s001.tif]

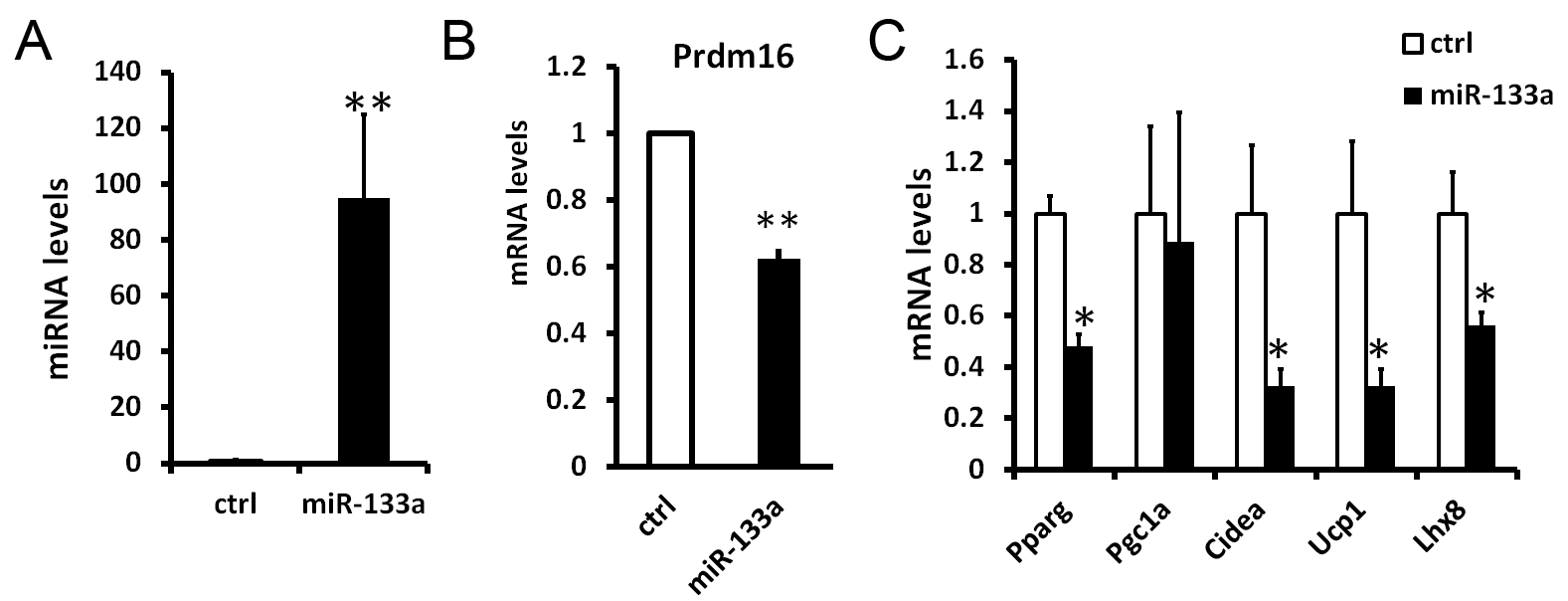

Supplement: Figure S2 — miR-133a inhibits adipocyte browning in SAT. SAT SVFs were transfected with synthetic miRNA133a by electroporation and cultured to confluence, followed by adipogenic induction and differentiation for 4 days each. (A–C) qPCR analysis of miR-133a and the brown markers after cells were differentiated. N = 3, *P<0.05, **P<0.01. (TIF) [file pgen.1003626.s002.tif]

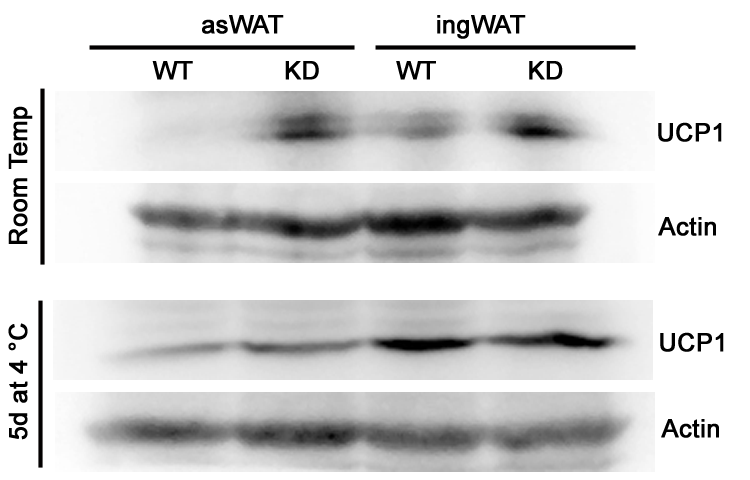

Supplement: Figure S3 — Knockdown of miR-133a upregulates UCP1 expression. Depots of asWAT and ingWAT were harvested from widltype (WT) and miR-133a knockdown (KO; miR-133a1−/−a2+/−) mice that were housed at room temperature or at 4°C for 5 days. Pictured are representative Western Blot images showing the relative expression levels of UCP1. Beta-Actin is used as internal control for protein input. (TIF) [file pgen.1003626.s003.tif]
